# Composition and regulation of the immune microenvironment of salivary gland in Sjögren’s syndrome

**DOI:** 10.3389/fimmu.2022.967304

**Published:** 2022-09-13

**Authors:** Zhen Tan, Li Wang, Xiaomei Li

**Affiliations:** Department of The First Affiliated Hospital of USTC, Division of Life Sciences and Medicine, University of Science and Technology of China, Hefei, China

**Keywords:** immune microenvironment, salivary gland, Sjögren’s syndrome, senility, chronic inflammation, hypoxia

## Abstract

Primary Sjögren’s syndrome (pSS) is a systemic autoimmune disease characterized by exocrine gland dysfunction and inflammation. Patients often have dry mouth and dry eye symptoms, which seriously affect their lives. Improving dry mouth and eye symptoms has become a common demand from patients. For this reason, researchers have conducted many studies on external secretory glands. In this paper, we summarize recent studies on the salivary glands of pSS patients from the perspective of the immune microenvironment. These studies showed that hypoxia, senescence, and chronic inflammation are the essential characteristics of the salivary gland immune microenvironment. In the SG of pSS, genes related to lymphocyte chemotaxis, antigen presentation, and lymphocyte activation are upregulated. Interferon (IFN)-related genes, DNA methylation, sRNA downregulation, and mitochondrial-related differentially expressed genes are also involved in forming the immune microenvironment of pSS, while multiple signaling pathways are involved in regulation. We further elucidated the regulation of the salivary gland immune microenvironment in pSS and relevant, targeted treatments.

## 1 Introduction

Primary Sjögren’s syndrome (pSS) is a systemic autoimmune disease that occurs mostly in middle-aged women and is characterized by impaired glandular function and the appearance of autoantibodies caused by infiltrating exocrine glands with lymphocytes, with an estimated prevalence of 0.3-3/1000 in the general population (1, 2). PSS is a heterogeneous disease; approximately 5% to 35% of the population has dry eye, approximately 20% of patients have dry mouth, and up to 34% of patients have parotid gland swelling (3–5). In addition to glandular involvement, arthritis occurs in approximately 50% of patients, lung involvement occurs in 9-12% of patients, skin lesions occur in 10% of patients, kidneys involvement occur in 5% of patients, and sensory neuropathy occurs in 10-25% of patients (6–8). In blood samples, 40%-75% of pSS patients have anti-RO/SSA, and 23%-52% have LA/SSB antibodies (9).

The etiology and pathogenesis of SS are still unclear, and it is generally believed that genetic susceptibility related to environmental factors is an important cause of the occurrence of Sjogren’s syndrome (10). Currently, genome-wide association studies on pSS have been completed, among which HLA genes have the strongest association signal (11). Epigenetic mechanisms such as DNA methylation, histone modification, and noncoding RNA play a role in the pathogenesis of pSS by regulating gene expression and may form a dynamic link between the genome and phenotypic expression. Bacteria and viruses are essential components of environmental factors. Bacteria can cause autoimmune diseases through various mechanisms, such as pathogen persistence, epitope spread, molecular mimicry, epigenetic changes, and Toll-like receptor activation. Type I IFN is a critical immune mediator involved in viral defense and immune response activation, which suggests the important role of viral infection in the pathogenesis of the disease. A recent analysis of the gene expression of SGECs showed that the IFN signaling pathway and genes involved in the immune response (HLA-DRA, IL-7, and B-cell activator receptor) in pSS were upregulated (12). Other studies found dysregulation of the IFN signaling pathway in SG and peripheral blood of some patients with SS (13), especially the upregulation of type I IFN-induced genes. Various factors, such as infection and hypoxia, induce the activation of SG epithelial cells, leading to lymphocyte infiltration (especially CD4+ T cells) and the release of inflammatory factors. New cell populations, such as follicular T cells, TH17 cells, dendritic (IFN-producing) cells, and B lymphocytes, gradually develop into B lymphocyte-dominated ectopic germinal centers (GCs) with autoantibody production.

The occurrence and development of pSS is a complex process involving many kinds of cells. The salivary gland, as the most commonly affected organ, has attracted increasing attention. Early SG lesions in pSS are rarely reported, which may be related to the delayed diagnosis of pSS. However, many studies have attempted to find targeted therapies for pSS by intervening in the inflammatory process of pSS. Among them, the improvement of dry mouth symptoms caused by impaired salivary gland function is an important goal of treatment. Autoantibodies in the salivary glands induce abnormal immune responses, which together with a large number of infiltrating inflammatory cells destroy normal salivary gland cells, atrophy of salivary gland cells and disappearance of salivary duct. As time goes on, the normal secretory function of salivary glands can be affected, resulting in dry mouth (14). Salivary gland cells, inflammatory cells, inflammatory mediators, autoantibodies and cytokines produced by each of them or by each other constitute the unique immune microenvironment of salivary gland cells. Changes in the immune microenvironment may cause changes in glandular function. Therefore, obtaining more salivary gland tissue samples, analyzing the potential differences between peripheral blood and salivary gland tissue, and further understanding the composition and regulation of the salivary gland immune microenvironment can help us find more targeted treatments for pSS xerostomia. In addition, the salivary gland immune microenvironment of pSS, a type of autoimmune epithelitis, can provide a model for the study of other autoimmune epithelitis (celiac disease, primary biliary cirrhosis, etc.).

## 2 Characteristics of the salivary gland microenvironment

The salivary gland microenvironment directly affects salivary gland secretion function and is very important for the occurrence and development of pSS. It generally has three characteristics: hypoxia, chronic inflammation, and senescence.

### 2.1 Hypoxia

Hypoxia is a state of reduced available oxygen caused by reduced blood flow, anemia, metabolic changes, and inflammation (15, 16). Hypoxia has been shown to accelerate cell apoptosis in the renal epithelium (17) while downregulating Cl- ion secretion in the intestinal epithelium, resulting in decreased epithelial fluid transport activity and destruction of tight connections between epithelial cells (18). Hypoxia can also lead to macrophage polarization, regulatory T-cell aggregation, and inhibitory T-cell maturation, leading to immune tolerance and tissue damage. A recent study of minor salivary glands in pSS patients found that hypoxia and IFN-related genes were closely associated with the expression of interleukin (IL)-21 signaling genes, which were significantly increased in pSS patients (19), suggesting a correlation between hypoxia and pSS morbidity. Hypoxia-inducible factor 1α (HIF1α), a transcription factor, is a major regulator of oxygen homeostasis and can be regarded as a hypoxia marker. HIF1α is also a key player in integrating the T-cell receptor (TCR) and cytokine receptor-mediated signals of CD4+ helper T cells (20). In addition, HIF1α enhances Th17 development through direct transcriptional activation of RAR-related orphan receptor gamma t (RORt) (21). This subpopulation is highly increased in salivary gland tissue of patients with SS and a mouse model of SS (22). Longyun Ye et al. cultured mouse submandibular glands (SMGs) *in vitro* and showed that hypoxia (5% O2) induced HIF-1α, glucose transporter 1 and VEGF expression, while BAY 87-2243-mediated HIF-1α inhibited salivary gland development (23). There are also differing viewpoints. Recent studies have shown that HIF1α expressed in epithelial cells protects against hypoxia-induced tight junction integrity loss and epithelial secretory function loss. The genotype and the allele of the HIF1A Pro582Ser polymorphism were associated with a reduced risk of pSS, suggesting that HIF1α activity may be involved in the development of pSS disease (24). Such different results may be related to different degrees of hypoxia. Antonela Romina Terrizzi et al. performed a comparative analysis of adult Wistar rats exposed to persistent or intermittent hypoxia over 90 days (25). The results suggested that salivary secretion decreased and prostaglandin E2 (PGE2) content increased in animals exposed to hypoxia. The persistent hypoxia group showed higher HIF-1α staining. This suggests that PGE2 plays a negative role during gland adaptation to hypoxia.

### 2.2 Chronic inflammation

Chronic inflammation is another feature of salivary gland involvement in pSS patients, mainly manifested by periductal lymphocytic infiltration. The lesions are mainly T and B lymphocytes, with a few monocytes, including macrophages, myeloid cells, plasmoid dendritic cells, and follicular dendritic cells (FDC) (26). The corresponding plasma cells attack normal tissues and organs, including salivary glands, and cause tissue damage when they produce autoantibodies. It has been reported that in a mouse salivary gland inflammation model, the degree of salivary gland inflammation is related to the titer of antinuclear antibodies (27). Some authors have described the presence of anti-Ro/SSA and anti-La/SSB autoantibodies in the saliva of pSS patients but no circulating antibodies in serum, suggesting that the salivary glands of pSS patients can specifically produce these autoantibodies (28). In addition to anti-Ro/SSA and anti-La/SSB autoantibodies, anti-salivary gland protein 1, anti-carboxylase 6, and anti-parotid secreted protein autoantibodies have also been reported, which recognize salivary gland- and lacrimal gland-specific antigens (29). Moreover, IL-2, IL-6, IL-21, BCL6, Foxp3, and other cytokines and transcription factors were detected in the salivary glands, which further proved the persistence of inflammation (30–33).

### 2.3 Senescence

Senescence is a permanent state of cell cycle arrest, with the upregulation of antiapoptotic pathways (34). Using organoid culture techniques, some researchers found that salivary gland progenitor cells (SGPCs) in pSS patients showed insufficient self-renewal ability and differential ability compared with the control group. The telomeres of pSS SGPCs were shorter than normal, suggesting the existence of an aging phenotype (35). In addition, p16+ expression increased in the basal striated duct cell (BSD) progenitor cell niche and the whole parotid epithelium, a marker of aging (36). Senescent cells express and secrete proinflammatory cytokines (i.e., senescence-related secretion phenotypes, SASP) that play a role in spreading senescence and promoting tissue inflammation (37).

In conclusion, pSS SGPCs tend to be senescent, and SASPs maintain the senescent SG microenvironment. The salivary function of pSS patients could not recover after the improvement of salivary gland inflammation, which proved the existence of senescence from another aspect. Mie Kurosawa et al. found an accumulation of senescence-associated T cells (SA-TS) in salivary glands of PSs model mice, which were involved in the pathogenesis of SS-associated sialadenitis through upregulation of the epithelial chemokine CXCL13 (38), and they may become another target for pSS treatment.

## 3 Composition of salivary gland immune microenvironment

### 3.1 Gland cells

First, let us review the SGs. The salivary gland is the general name for the exocrine gland opening in the mouth through the duct because its secretions are discharged into the mouth and mixed into saliva. There are three pairs of SGs: parotid, sublingual and submandibular, and the largest pair is the parotid gland. SGs are composed of repeatedly branching ducts and terminal acinus forming the gland parenchyma. The acinar is divided into serous, mucinous, and mixed acinar, producing and secreting watery or mucus-rich saliva from serous and mucinous acinar cells. The secreted saliva passes through intercalated ducts into striate tubes of basal cells and lumen cells and finally into the mouth through larger excretory ducts. Luminal striated duct cells, basal striated duct cells, intercalated ducts, acinar cells, and myoepithelial cells constitute the salivary gland’s epithelial cells (SGECs). Salivary progenitor cells reside in striatal canals and proliferate and differentiate to maintain gland homeostasis.

SGECs are not only a critical immune target of pSS but also play an essential immune function in the pathogenesis of pSS, mediating the initiation and persistence of inflammation and autoimmune response. Human pSS SG epithelial cells show increased proapoptotic molecules (such as Fas and Bax) and decreased antiapoptotic molecules (Bcl-2) compared with healthy individuals (39–41). Endoplasmic reticulum stress leads to autophagy and apoptosis, which may lead to redistribution of Ro/SSA and La/SSB autoantigens, initially on the cell surface and eventually in apoptotic blisters (42). These autoantigens are upregulated in pSS SGECs and regulated by the TLR/IFN TYPE I signaling pathway (43). SGECs are not only important sources of pSS autoantigens Ro/SSA and La/SSB but also express MHC class I and II and T-cell costimulatory molecules (CD80/CD86), enabling them to function as autoantigen presenting cells (44–46). SGECs have been shown to express not only virus-associated toll-like receptors (TLRs) (3 and 7) but also bacterial infection-associated TLRs (1, 2, 4) (47). SGECs bind to multiple pathogen-associated molecular patterns (PAMPs) through the expression of TLRs 1, 2, 3, 4, and 7. In addition to the B-cell activator CD40, SGECs also express CXCL10, CXCL12, CXCL13, IFNα, IFNβ, IFNλ, TNFα, and other receptors. They also produce a variety of cytokines/chemokines under various stimulus conditions, including IL-18, IL-21, IL-1, IL-6, TNFα, B-cell activating factor (BAFF), CXCL-10, CXCL-12, CXCL-13, and CCL-21 (47–59). IL-6 and the costimulatory molecule ICOSL contribute to follicle-assisted T-cell induction, which is critical for B-cell activation and differentiation (60). IFNλ stimulation of SG epithelial cells also induces the expression of BAFF and CXCL10, suggesting that type III IFN plays a role in developing SG pathology in pSS (61).

Abundant evidence suggests that SGECs can drive the activation, differentiation, and survival of B cells through direct interaction and cytokine production and promote the pathogenesis of SS (12, 62, 63). *In vitro*, culture results showed that SGECs from pSS patients promoted the differentiation of B cells into mature B-cell phenotypes and improved the survival rate (12, 63). SGECs may also indirectly induce B-cell differentiation. SGECs have been reported to promote T follicular helper cell differentiation and IL-21 production (60), which may further enhance B-cell hyperactivity in salivary glands in pSS patients. In the salivary glands of pSS patients, high levels of CXCL12 were detected in ductal epithelial cells (64), and CXCL12 expression and IL-6 were associated with high focusing scores and high levels of CD138+ plasma cell infiltration (51). Riviere et al. showed the presence of IL-7/IFNγ amplification loops involving SGEC and T cells in primary SS (65). They stimulated primary cultures of SGECs from control and primary SS patients with poly (I-C), interferon α, or interferon γ. SG explants were cultured with an anti-IL-7 receptor (IL-7R) antagonist antibody (OSE-127) for 4 days, and transcriptome analysis was performed using the NanoString platform. The results suggested that the expression of IL7R was decreased in T cells. Il-7 is secreted by SGECs stimulated by poly (I-C), IFNα, or IFNγ. IL-7 stimulation increases T-cell activation and IFNγ secretion. Transcriptome analysis of SG explants showed a correlation between IL7 and IFN expression, and explants cultured with anti-IL-7R antibodies showed reduced IFN-stimulated gene expression. These results indicate the presence of IL-7/IFNγ amplification loops involving SGEC and T cells in primary SS. Il-7 is secreted by the SGEC stimulated by type I or TYPE II IFN, which in turn activates T cells that secrete type II IFN.

The stromal component of the salivary glands is composed of mesenchymal stromal cells (MSCs), which provide tissue-homeostatic properties, including regeneration, repair, and immune regulation. It has been shown that human bone marrow mesenchymal stem cells (hMSCs) cocultured with purified immune cell subpopulations alter the cytokine secretion profile of dendritic cells (DC), primary and effector T cells (Th1 and Th2), and natural killer cells (NK) to induce a more anti-inflammatory or tolerant phenotype (66). This suggests that MSCs may reduce inflammation by acting as immunomodulators and promoting tissue regeneration. In recent studies, IFNγ stimulated cultured resident MSG-derived MSCs (MSG-Mscs) isolated from the small salivary glands of pSS patients, and the protein levels of indoleamine 2,3-dioxygenase (IDO), programmed death ligand 1 (PD-L1), and intercellular adhesion marker 1 (ICAM-1) increased. These results suggest that MSG-Mscs have normal immunomodulatory functions in small salivary glands. In addition, MSG-Mscs inhibited T-cell proliferation in a dose-dependent manner and were not associated with 17-β-estradiol exposure (67). In addition, follicular dendritic cells (FDCs) are stromal cells located in primary follicles and germinal centers (GCs) of secondary and tertiary lymphoid organs and have the unique ability to retain natural antigens in B-cell follicles for several months (68).

### 3.2 Multiple participating cells

With the progression of epithelial cell activation and disease, new cells appear in the salivary gland, such as follicle T cells and Th17 cells, dendritic cells (producing IFN), macrophages (MFs), natural killer cells, and B lymphocytes (T cells are usually found in mild lesions, and B cells and MFs dominate in the most severe lesions). Furthermore, they gradually develop into ectopic germinal centers (69, 70).

#### 3.2.1 B cells

There are many different types of B cells in salivary gland tissue. FcRL4+B cells were found in or near the ductal epithelium of the inflammatory salivary gland tissue of pSS. FcRL4 is closely related to lymphoma and is expressed in almost all MUCOSAL-associated lymphoid tissue (MALT) B-cell lymphomas associated with pSS, especially in the parotid gland. RNA sequencing of FcRL4+ B cells isolated from parotid cell suspensions from pSS patients showed that FcRL4+ B cells were not enriched in the blood of pSS patients compared with non-SS-sicCA patients, but these cells generally displayed a proinflammatory phenotype. Genes encoding CD11c (ITGAX), T-BET (TBX21), TACI (TNFRSF13B), Src tyrosine kinase and NFκB pathway-related genes were significantly upregulated in glandular FcRL4+B cells compared with FCRL4-B cells. Therefore, FcRL4+B cells in pSS exhibit many characteristics of chronic activation and proinflammatory B cells (71). Some researchers found that through immunohistochemistry and mRNA analysis, the expression level of FcRL4 mRNA in parotid MALT lymphoma was increased compared with the parotid tissue of pSS patients without lymphoma, which may explain why MALT lymphoma in pSS patients preferentially occurred in this specific site (15). In addition, MZB cells were detected in saliva and lacrimal glands in both patients with salivary gland disease and mouse models. The C57BL/6. Nod-aec1aec2 mouse model, as well as several SS gene knockout mouse models, showed that B lymphocytes, especially peripheral zone B (MZB) cells, are necessary for the development of clinical manifestations and pathogenesis, although destruction of lacrimal and salivary gland cells involves a typical T-cell-mediated autoimmune response. Peck et al., through *in vitro* temporal global RNA transcriptomic analysis, showed that MZB cells from C57BL/6. Nod-aec1aec2 mice were recruited by the upregulated Cxcl13 chemokine to the exocrine gland, where they recognized complement-modified antigens through their sphingosin-1-phosphate and B-cell receptors (72). BAFF transgenic (TG) mice developed autoimmune diseases characterized by autoantibody production, leading to salivary gland destruction (salivary adenitis), which was associated with enlargement of the B-cell compartment in the marginal region (MZ) and abnormal presence of MZ-like B cells in blood and inflamed salivary glands (15). In the IL14aTG mouse model, elimination of MZB from mice by B-cell-specific deletion of RBP-J resulted in complete elimination of all SS disease manifestations (73). Daridon and others, through classification and reverse transcription polymerase chain reaction analysis of salivary gland specimens in the presence of B cells and its polyclonal form in 18 patients, found that pSS patients with heterotopic salivary glands in GC sample structure transition - 2 B-cell amplification, locally produce autoantibodies, which may help and influence subsequent epithelial damage (74). Immunoglobulin rearrangement in single parotid B cells isolated from the parotid gland was analyzed by fluorescence-activated cell sorting, and the results showed that compared with peripheral blood, most parotid B cells in pSS showed the mutant status and phenotype of memory B cells, which accumulated in the salivary glands of pSS patients (75). Hansen et al. analyzed chemokine receptor expression in CD27- naive and CD27+ memory B cells from primary SS patients and healthy controls using flow cytometry, single-cell reverse transcription polymerase chain reaction (RT−PCR), and migration assays. The results showed that CD27+ memory B cells overexpressed the chemokine receptors CXCR4 and CXCR5, which may promote the infiltration of memory B cells into inflammatory glands through the chemokine receptors CXCL12 and CXCL13 from epithelial cells (76). Similar to transition B cells, CD27+ memory B cells seem to promote the formation of ectopic GC-like structures in the exocrine glands of pSS patients (77). Skarstein et al. found that in pSS patients, the increase in CD138+ plasma cells and CD20+ B cells is associated with fat infiltration and focal infiltration, suggesting that they are actively involved in promoting inflammation (78). Szyszko et al. performed single, double, and triple immunohistochemical and immunofluorescence staining of small salivary gland tissues from pSS, chronic inflammatory, and normal subjects, suggesting that plasma cells were located near CXCL12- and IL-6-expressing cells. A salivary gland environment with a high focus score provided a critical factor for plasma cell survival (51). In addition, Cui et al. developed an enzyme-linked immunosorbent assay for autoantibodies with good quantitative ability and found that the expression levels of saliva anti-Cofilin-1, anti-α enolase and anti-RGI2 in parotid gland tissues of pSS/MALT patients were significantly higher than those of healthy controls (79). These results suggest the promoting role of plasma cells in MALT lymphoma.

#### 3.2.2 T cells

In the salivary glands, T cells mainly assist B cells. Th1 cells are believed to play a major role in pSS and are the most relevant CD4+ cell population infiltrating inflammatory SGs (80). Th2 cytokine levels are closely associated with SG lymphocyte infiltration (30). Th17 cells also play a key role in pSS. Th17 cells in SGs can develop into Th17.1 cells and produce IL-17 and IFN-γ, which are involved in the pathogenesis of pSS (81). It has been reported that Tfh cells selectively accumulate in the SGs of pSS patients (82, 83). Tfh cells appear as a unique subpopulation of CD4+ T helper cells that promote the development and activation of B cells. Tfh cells express CXCR5, which migrates and localizes in B-cell follicles and induces the expression of T-cell costimulatory (ICOS) molecules, coinhibitory programmed cell death protein-1 (PD-1), and the transcription factor Bcl6 (84). Tfh cells release large amounts of IL-21, a key cytokine that activates the molecular mechanism of somatic excessive mutation and analog switching of B cells (85, 86). Another subpopulation of CD4+ T cells, follicular regulatory T cells (Tfr), also express CXCR5 but have the typical inhibitory function of regulatory T cells, negatively regulating GC responses to prevent abnormal GC responses (87). In addition, pSS patients showed a high degree of infiltration of pathogenic T peripheral helper cells (Tph) in SGs, which lacked typical Tfh markers such as CXCR5 and Bcl6 but could assist homologous B cells through IL-21 and CD40-L (82, 88). By studying the peripheral blood of pSS patients, Dupre et al. found that Tfh and Tph were amplified in the peripheral blood of patients and correlated with disease activity and B-cell marker (RF and anti-SSB) levels (89). Pontarini et al. performed transcriptome (microarray and quantitative PCR) analysis, FACS T-cell immunophenotyping and intracellular cytokine detection, polychromatic immunofluorescence microscopy and *in situ* hybridization. It was found that damaged CD4+CD45RO+ICOS+PD1+ cells selectively infiltrated ELS+ tissues in SG and amplified abnormally in parotid malt-L. In ELS+SG and MALT-L parotid glands, the traditional CXCR5+CD4+PD1+ICOS+ FOXP3-TFH cell population and the uniquely enlarged CXCR5-CD4+PD1HIICOS+ Foxp3-TPH cell population showed frequent IL-21/interferon dual production. The results highlight Tfh and Tph cells and IL-21 and ICOS costimulation pathways as key pathogenic factors in SS immunopathology (90). Cytotoxic T lymphocytes (CTLs) can specifically recognize and lyse their targets. Recently, antigen-specific cytotoxicity expressed by cytotoxic T cells *in vitro* and *in vivo* has been shown to be Fas based (91). Kong et al. used immunohistochemical staining and reverse transcription polymerase chain reaction *in situ* to detect the expression of Fas and Fas ligand (FasL) in salivary gland biopsy materials and evaluated the DNA fragments in apoptotic cells by enzymatic incorporation of labeled nucleotide (digoxin -dUTP). The results showed that the acinar epithelial cells of SS were Fas+ and FasL+, and the cells died by apoptosis. Fas+ and Bcl-2+ were the dominant infiltrating lymphocytes in SS, and FasL was expressed in a few lymphocytes. *In situ* detection of apoptosis showed minimal cell death of lymphocytes, especially in dense periductal lesions. These results suggest that the Fas pathway may be an important mechanism of SS gland destruction (39).

#### 3.2.3 Other cells

Dendritic cells (DCs) can be divided into antigen presenting myeloid DCs (MDCs), which are effector cells, and plasma cell DCs (PDCs), which mainly produce type I interferon. Among them, plasmacytoid dendritic cells (PDCs) produce type I interferon (IFN) and contribute to the pathogenesis of various autoimmune diseases. PDC and type I IFN activity are elevated in the salivary glands of SS patients. Zhou et al. applied pdC-consuming anti-BST2/CD317 antibodies to female NOD mice aged 4 to 7 weeks at the early stage of SS and assessed the pathology of SS at 10 weeks of age. The results suggested that PDC treatment inhibited the development of inflammation and secretory dysfunction of SMG and significantly reduced the number of type I interferon mRNA, total white blood cells, T lymphocytes and B lymphocytes in SMG. This suggests a role for PDC in the pathogenesis of pSS (92). In patients with pSS, immature myeloid dendritic cells (DCS) are reduced in the blood, and mature myeloid dendritic cells accumulate in the salivary glands. As the duration of pSS syndrome increases, the reduction in myeloid dendritic cells in the blood spontaneously recovers. Myeloid DCs may play an important role in the pathogenesis of pSS by initiating the helper T-cell immune response (93).

Fibroblasts are an extremely heterogeneous population of cells with a spindle shape, oval nuclei, and the ability to adhere to collagen fibers. In addition to synthesizing and reshaping the structure and function of the extracellular matrix, they also have the ability to secrete cytokines, chemokines and growth factors. Furthermore, immune fibroblasts affect the homeostasis of immune cells, and are one of the important stromal cells constituting the tertiary lymphoid structures (TLS) (94). Nayar et al. showed that immune fibroblast activation and expansion were observed during TLS formation in wild-type (WT) mice that induced salivary gland inflammation. In Dm2 mice, the loss of PDPN^+^/FAP^+^ fibroblasts disrupted the establishment of TLS and impaired the establishment of local pathology. Meanwhile, in salivary glands of pSS patients, the phenotype and proliferation of TLS immune fibroblasts are regulated by IL-13 and IL-22 (95). In addition, Korsunsky et al. performed single-cell RNA sequencing of fibroblasts and found that CXCL10^+^CCL19^+^ immune-interacting and SPARC^+^COL3A1^+^ vascular-interacting fibroblasts were expanded in a variety of inflammatory tissues (salivary glands of pSS, synovium of RA, colon of ulcerative colitis, etc.) (96). In ulcerative colitis, fibroblasts are the main source of IL-6, and many cytokines and inflammatory mediators have been found to significantly induce IL-6 expression in fibroblasts, including TNF, IL-17, IL-1, LPS, and IFN (97).

### 3.3 Extracellular matrix

SS is essentially a kind of epithelial inflammation, and the integrity, structure, and function of epithelial cells largely depend on the homeostasis of the extracellular matrix (ECM). The ECM is a network of many components, including fibrin, glycosaminoglycan, growth factor, protease, and inhibitors. An increasing number of studies have shown that changes in the morphology and function of acini and ducts, accompanied by the degradation and remodeling of ECM, are critical events in salivary gland changes in pSS patients. ECM not only supports glandular cells, but its components are also important components of damage-related molecular patterns (DAMPs). DAMPs are potential endogenous inflammatory sources that drive autoimmunity by activating pattern recognition receptors (98). When the glandular tissue of pSS patients is damaged by internal and external environmental factors, the ECM releases soluble DAMPs (Biglycan, decorin, etc.) under the action of matrix metalloproteinases (MMPs). Soluble DAMPs activate homologous receptors that mediate inflammation (e.g., MyD88-dependent TLRs), leading to aseptic inflammation and enhancing pathogen-mediated inflammation (99–101). The salivary mucins MUC1, MUC7, and MUC5B secreted into the intercellular space have been reported to activate proinflammatory molecules and Toll-like receptors, which are also involved in inflammatory responses (99). Aberrant decorin levels (DCN) induce damage to human salivary gland epithelial cells and the polarization of macrophages (102). The increased level of DCN in the parotid gland of pSS patients was positively correlated with several chemokines (CXCL13, CXCL9, and CCL20), IL-1ß, and caspase3 but negatively correlated with the proliferation-related gene MKI67. DCN induces apoptosis of A253 cells and differentiation of macrophages into the M1 phenotype, which is characterized by the expression of proinflammatory cytokines (102).

## 4 Special structure

**Figure 1:**
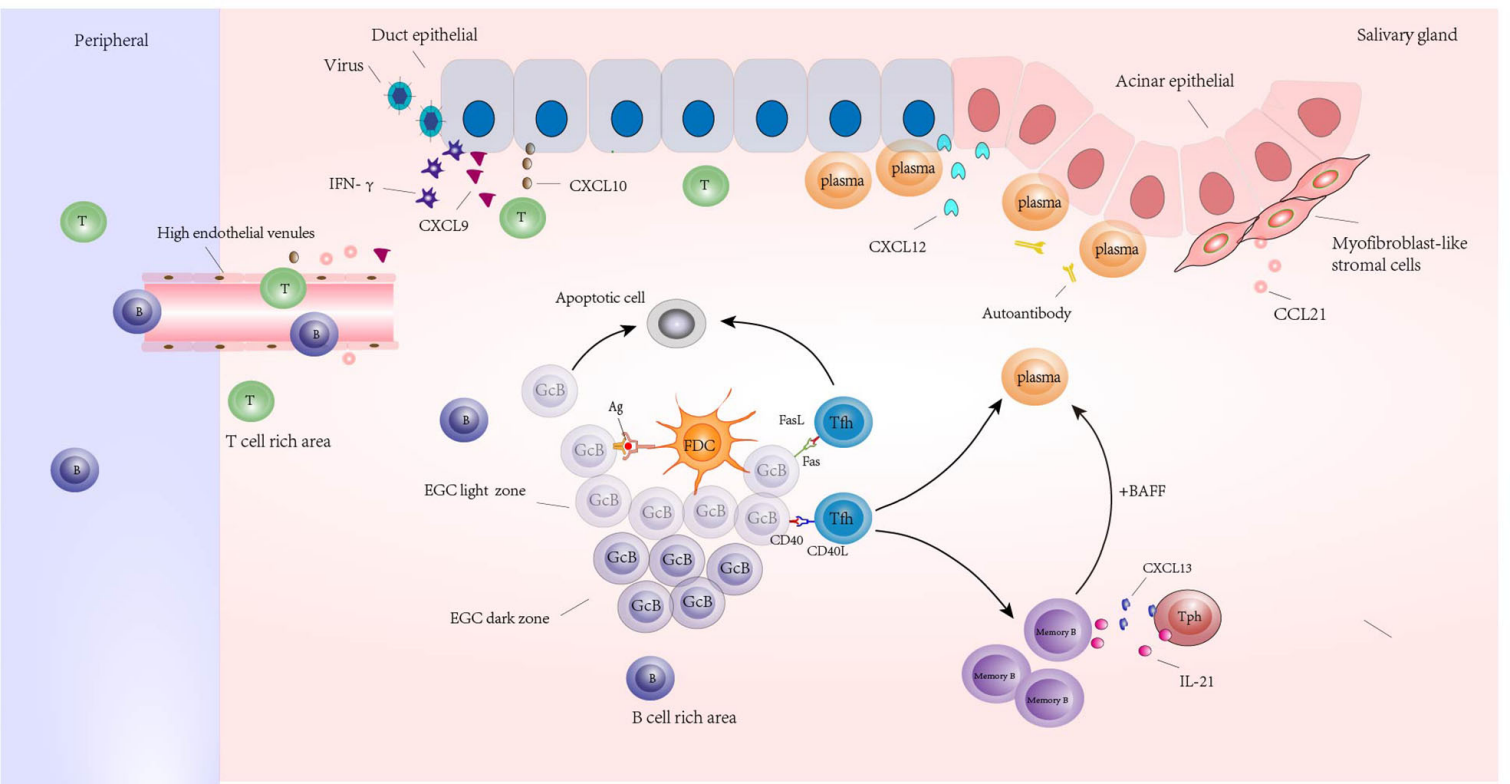
Ectopic lymphoid structures (ELSs) formed around the glands in the salivary glands of a patient with Sjogren’s syndrome. Its core is the ectopic germinal center (EGC), including the bright and dark regions; Germinal center B cells (GcB) were screened by follicular dendritic cells (FDC) in the bright region. The GcB cells that did not receive antigen presentation were apoptotic, and the GcB cells that received antigen presentation were transformed into memory B cells and plasma cells under the assistance of follicular helper T cells (Tfh) or were regulated by Tfh cells to undergo apoptosis. Under the regulation of IL-21 and CXCL13 produced by Tph, memory B cells were transformed into plasma cells under the combined action of BAFF and other factors. Plasma cells infiltrated around ducts and acinar epithelial cells expressing CXCL12 and produced autoantibodies. There are B cell rich areas and T cell rich areas around EGC. High endothelial venules (HEVs) appear in the periphery of lymphoid aggregates in T cell-rich areas, and HEV formation is influenced by CCL21 produced by myofibroblast-like stromal cells; Under the stimulation of virus and other inducements, tissues expressed IFN-γ and induced the release of CXCL9 and CXCL10; CXCL9 and CXCL10 promote the accumulation of peripheral T and B lymphocytes that enter the gland through HEV to the site of inflammation.

The salivary glands of pSS patients are characterized by chronic inflammation, and the lesions are mainly composed of T and B lymphocytes (26). In the initial stage of disease, lymphoid tissue initiator or inducer (LTi) cells, induced by precursors such as Epstein-Barr virus or cytomegalovirus, produce lymphotoxin, which promotes the expression of NF-κB signals in lymphoid tissue organizer cells by lymphotoxin-β receptors. This results in the enhanced expression of homeostatic chemokines and cytokines (CXCL13, CCL19, CCL21, RANKL, IL-17 and IL-22) (103, 104). At the same time, the virus induced the expression of interferon-γ and stimulated the expression of CXCL9 and CXCL10 in ductal epithelium. These cytokines and chemokines are involved in attracting T lymphocytes, B lymphocytes, and other immune cells to the site of inflammation and promote the formation and maintenance of organized lymphoid tissue (105–107). In addition, prolonged gland activation leads to the formation of FDC networks and the separation of T and B cells, and finally to the formation of ectopic lymphoid structures (ELS) with ectopic germinal centers as the core, T and B cell separation areas surrounding, and the formation of high endothelial venules (HEVs) as exchange channels with peripheral blood lymphocytes (103, 104, 108). The germinal center consists of a light zone and a dark zone. There are rapidly proliferating central blasts in the dark zone, and their Ig variable region genes can undergo somatic hypermutation, thereby protecting them from apoptosis. In the light zone, the central cells transformed from the central blast cells competitively bind to the antigen presented by FDC. The central B cells with high affinity BCR bound to the antigen were positively selected, and the cells that did not receive the antigen underwent apoptosis. Tfh cells regulate the apoptosis of positively selected central B cells or further development into memory B cells or plasma cells through Fas-FasL and CD40-CD40L (109). Tph further induces chemotaxis and conversion of memory B cells into plasma cells by producing IL-21 and CXCL13. Initiation and maintenance of ELS in pSS requires ectopic expression of lymphoid chemokines, including CXCL12, CXCL13, CCL19, and CCL21. These chemokines regulate lymphocyte trafficking and tissue localization by interacting with their unique receptors CXCR4 (for CXCL12), CXCR5(for CXCL13), and CCR7(for CCL19 and CCL21). CXCL12 is mainly produced by follicles and ducts, and its receptor is expressed in plasma cells (110). CXCL13 is mainly produced by stromal cells, memory CD4+ T cells, and non-immune cells (ductal epithelial and endothelial cells) in the FDC network. CCL21 is released from myofibroblast-like stromal cells and is closely associated with HEV formation (**Figure 1**).

## 5 Regulation of the immune microenvironment

### 5.1 Gene regulation

#### 5.1.1 Genes

In the SG of pSS, upregulated genes are associated with lymphocyte chemotaxis, including IFN-induced chemokines such as CXCL10, and lymphocyte activation, such as TCR β-sites, which play a central role in T-cell activation. The MHC genes HLA-DR and HLA-DQ, which are related to antigen presentation, were also highly expressed in pSS. CXCL13 and CD3D genes were expressed in >90% of pSS patients and <10% of controls, confirming lymphocyte chemotaxis and activation in the SG of pSS patients. Lymphocyte-β (LTβ) is involved in ELS formation in inflammatory tissues and is one of the top 50 differentially expressed genes (DEGs) (111). Shimoyama et al., using single nucleotide polymorphism (SNP)-specific sequencing, found that the risk allele of human salivary gland GTF2I SNPs increased GTF2I expression and enhanced nuclear factor κB (NFκB) activation in human salivary gland cells *via* the NFκB P65 subunit (112). Inamo et al. used microarray technology to detect peripheral blood B cells of pSS patients and healthy controls and identified LINC00487 and SOX4 as key genes of B-cell disorder in pSS patients by the WGCNA algorithm (113). Many type I IFN genes associated with the response to viral infection were found in the first 200 genes with increased expression in pSS (114). These include IFNα-inducible protein 27, 9-27 (IFITM1), IFN stimulates gene 12 (ISG12), GBP2, and IFN regulator 8 (IRF8). Furthermore, the EBV-induced ligand (CCL19) and its receptor CCR7 genes were upregulated in pSS SGs. These two genes are involved in the activation of B and T cells.

In addition to infection, oxidative stress is an important cause of Sjögren’s syndrome, and reactive oxygen species (ROS) in oxidative stress mainly come from mitochondria. Mitochondria are organelles necessary to maintain homeostasis in cells, and their function is maintained by dynamic fine-tuning. Damaged mitochondria produce more ROS than healthy mitochondria. Changes in mitochondrial-endoplasmic reticulum contact sites (MERCs) can increase inflammatory signals and regulate stress responses and intracellular homeostasis, ultimately affecting cell fate (115). Damaged mitochondria produced more reactive oxygen species than healthy mitochondria, and the presence of mitochondria in pSS salivary gland cells resulted in severe ultrastructural changes (115). Recent scientific studies have shown that mitochondria-related differentially expressed genes (CD38, CMPK2, TBC1D9, and PYCR1) are closely related to the immune cell infiltration of salivary glands in pSS patients through real-time quantitative PCR (116).

#### 5.1.2 Epigenetics

Moreover, epigenetics, which includes DNA methylation, noncoding RNA and histone modifications, are involved in the regulation of inflammatory signals in pSS.

##### 5.1.2.1 DNA methylation

At present, there are many literature reports on DNA methylation and pSS (117). DNA methylation is catalyzed by DNA methyltransferase (DNMT) and refers to the presence of methyl radicals (methyl) in CpG dinucleotides from 5-methylcytosine (5-MC). A study on salivary gland methylation showed that the level of salivary gland epithelial cell methylation in pSS patients was lower than that in healthy individuals through DNA global methylation and real-time PCR detection (118). Coculture of human salivary gland cell lines established from irradiated tumor epithelial duct cells and Ramos human B-cell lines suggested that DNA demethylation is associated with lymphocyte infiltration, especially B-cell infiltration, and that the protein kinase C δ - extracellular signal-regulated kinase DNA methyltransferase 1 pathway may be involved in this phenomenon (118). However, DNA methylation is an epigenetic mechanism that includes the adjustment of gene expression and is heritable and reversible without modifying the DNA sequence. This provides another direction for the treatment of pSS.

##### 5.1.2.2 MiRNAs

MicroRNAs (miRNAs) are small noncoding RNAs (sRNAs) that alter gene expression by binding to target messenger RNAs (mRNAs) and inhibiting translation. A researcher (119) conducted sRNA analysis based on global next-generation sequencing (NGS) for pSS labial salivary glands (LSGs) and sicca control groups, and the results suggested that 30% of sRNA in pSS LSG was miRNA, and the miRNA with the most significant change was HSA-Mir-181D-5p compared with the control group. The mRNA level of TNF-α, a direct target of HSA-miR-181D-5p, was significantly increased and negatively correlated with the presence of HSA-miR-181D-5p. Downregulation of HSA-miR-181D-5p in the LSG of SS patients may promote the adenoinflammatory environment by deregulating its direct target TNF-α.

##### 5.1.2.3 Histones

The N-terminal tail of histones protrudes from the nucleosome and is subject to various covalent posttranslational modifications, including acetylation, methylation, phosphorylation, ADP ribosylation, protein conjugation, β-N-acetylglucosamination, deimination/citrullination and ubiquitination/sumoylation. A variety of enzymes are involved in histone modification, such as histone deacetylases (HDACs), histone acetyl transferases (HATs) and histone methyl transferases (HMTs). It has been widely reported that HDACs are involved in immune reactions. HDAC4 negatively regulates the polarization of naive CD4+ T cells toward the Th17 phenotype (120), while HDAC6 induces IL-13 expression through AP-1, leading to the polarization of M2 macrophages (121). Sirtuin 2 (SIRT2), a member of the NAD+-dependent histone deacetylase family, promotes the deacetylation of p70S6K, activates the mTORC1/HIF-1α/RORγ T pathway, inhibits the production of IL-2 by CD4+ T cells, and promotes their differentiation into Th17 cells (122). Histone deacetylase inhibitors (HDACi) modulate the inhibitory T lymphocyte subsets of regulatory T cells (Tregs) and enhance FoxP3 acetylation, thereby protecting transcription factors from proteasome degradation. *In vitro* T-cell culture experiments in mice showed that HDACi reduced the proliferation of effector T cells and enhanced the inhibitory function of Tregs in coculture with effector T cells (123). HDAC6 inhibitors inhibit Th17-cell differentiation through the PKM2/STAT3 axis (124), while trastatin A (TSA), an HDAC inhibitor, inhibits dendritic cell maturation through downregulation of NF-κB (P65) (125). HDAC also plays an important role in B cells. Studies have shown that the development and survival of plasma cells depend on HDAC11, and the number of plasma cells in the peripheral blood of mice lacking HDAC11 is significantly reduced. In B cells lacking functional HDAC11, the differentiation of plasma cells *in vitro* is blocked, and HDAC11 is involved in the deacetylation of IRF4 at lysine 103 (126).

### 5.2 Signal transduction

#### 5.2.1 BAFF

BAFF is a crucial cytokine of B cells, promoting B-cell maturation, proliferation, and survival. In pSS patients, salivary gland epithelial cells and innate immune cells can secrete BAFF (127, 128). Elevated LEVELS of BAFF were detected in salivary glands (127). Epithelial cells promote B-cell activation through BAFF, and BAFF may also promote epithelial cell survival through a member of the TNF receptor superfamily 13C (also known as the BAFF receptor) (129). BAFF is induced by type I and type II IFN (130). Therefore, TNFSF13B, encoding BAFF, can be considered the gene stimulated by IFN. IFN regulatory factors (IRFs) control the transcription of TNFSF13B: IRF1 and IRF2 are induction factors of TNFSF13B transcription, while IRF4 and IRF8 are inhibition factors (131). TNFSF13B transgenic mice overexpressing BAFF first developed a lupus-like phenotype and then acquired pSS characteristics, including reduced salivation. This finding supports the role of BAFF in promoting the pathogenesis of pSS.

#### 5.2.2 EGF

The SG epithelium relies on a variety of signaling pathways to maintain homeostasis. One pathway is epithelial growth factor (EGF) signal transduction. For TLR signal transduction, EGF receptor (EGFR) activation has also been required for TLR3 signal transduction in the epithelial cell line and TLR-4-mediated downstream NFκB pathway activation, although in the cancer cell model system (132, 133). Conversely, TLR4 signaling also activates EGFR signaling, epithelial cell proliferation, and EGFR ligand expression (134).

#### 5.2.3 NFκB

The NFκB family is a group of transcription factors that activate a range of inflammatory downstream targets when they translocate to the nucleus. The NFκB pathway has been well demonstrated to be active in pSS SGECs. Phosphorylated IKKϵ (pIKKϵ), pIκBα, and pNFκB were highly expressed in the ductal epithelium of small SGs in pSS patients (135). In pSS SGECs, the expression of the NFκB inhibitor IκBα was significantly lower than that in healthy controls (135, 136), and IκBα inhibited NFκB activity by masking nuclear localization signals. Stimulation of TLR2 receptors in SGECs induces IL-2 production through the NFκB pathway in pSS SGECs (137, 138). In the human SG cell line, IL-6 upregulation is regulated by a set of pathways, including NFκB (139). Knockout of the natural NFκB inhibitor A20 in K14+ epithelial cells (thereby activating the constitutive pathway) is sufficient to trigger the initial stages of pSS, including reduced saliva production and lymphocyte invasion of SGs (54).

#### 5.2.4 EMT

The transdifferentiation of epithelial cells into motile mesenchymal cells, a process known as epithelial-mesenchymal transformation (EMT), is essential for development, wound healing, and stem cell behavior and contributes pathologically to fibrosis and cancer progression (140). In primary epithelial tumors, the interaction between cells and the extracellular matrix is reshaped during EMT, resulting in the separation of epithelial cells from each other and the underlying basement membrane and the formation of migratory mesenchymal cells that migrate to different sites with blood flow (141). In pSS, the EMT process mainly involves fibrosis of epithelial cells. During fibrosis, EMT responds to the triggering of TGF-β1, and TGF-β family receptors mediate intracellular signaling cascades that activate SMAD family members through SMAD 2/3 phosphorylation (142). Sisto et al. exposed cultures of healthy salivary gland epithelial cells (SGECs) from healthy donors to TGF-β1 treatment. Semiquantitative RT−PCR, quantitative real-time PCR and Western blot analysis were performed to compare the related gene and protein levels of Smad2/3/4, Snail, e-cadherin, Vimentin and type I collagen (143). They observed higher expression of SMAD2, 3, and 4 and Snail in TGF-β 1-exposed SGECs than in untreated healthy SGECs at both the genetic and protein levels. Snail is the transcriptional repressor and promoter of EMT. Furthermore, compared with untreated SGEC, we found a significant decrease in the epithelial phenotypic marker e-cadherin and a significant increase in the mesenchymal phenotypic marker vimentin and type I collagen in the TGF-β 1-treated samples. This finding suggests that TGF-β1 induces EMT through the TGF-β1/SMAD/Snail signaling pathway, further confirming the existence of EMT in SGs. Concurrent use of the specific TGF-β1 inhibitor SB-431542 in healthy SGECs treated with TGF-β1 significantly reduced the fibrosis markers vimentin and type I collagen, while the epithelial marker e-cadherin returned to levels similar to those of untreated healthy SGECs. This further confirms that TGF-β1 plays an important role in EMT-dependent fibrosis. IL-17 and IL-22 play an important role in EMT. Through the study of salivary glands of pSS patients, researchers found that the expression of the epithelial marker E-cadherin was negatively correlated with the increase in tissue inflammation in pSS SG specimens, while the expression of mesenchymal vimentin and type I collagen was positively correlated. At the same time, they assessed the effect of IL-17 and IL-22 treatment on EMT-dependent SG fibrosis in primary human salivary gland epithelial cells (SGECs) isolated from healthy subjects. The results suggest that vimentin and type I collagen are upregulated after interleukin treatment, while e-cadherin expression is decreased, and the cooperation between IL-17 and IL-22 is required to induce EMT (144).

#### 5.2.5 JAK/STAT

Recent results show that IFN-γ specifically inhibits the early steps of TGF-β-induced SMAD3 activation through the JAK/STAT pathway while inducing a rapid increase in SMAD7 expression (145, 146). SMAD7 binds to the TFG-β-receptor complex to inhibit TGF-β-mediated phosphorylation of SMAD3 and block TGF-β signaling (35), promoting SG precursor cell differentiation and saliva production (147). Pringle et al. recently demonstrated that SG progenitor cells respond to proinflammatory cytokines through proliferation and apparent cell death, likely through the JAK/STAT signaling pathway, suggesting that JAK/STAT signaling pathway inhibitors may interfere with SG epithelial homeostasis (35).

#### 5.2.6 IFN

Microarray and real-time quantitative polymerase chain reaction (RT-qPCR) studies showed that IFN-stimulating genes were significantly upregulated in small salivary glands (MSG) in pSS patients compared with healthy controls (148, 149). Studies have shown that specific type I IFN-associated transcripts (IFIT-3) and type II IFN-associated transcripts (GFP-2) are expressed in MSGs, and IFIT-3 is mainly located in the duct epithelial cells of salivary glands. Gbp-2 is simultaneously located in ductal epithelial cells in lymphocyte aggregates and inflammatory cell infiltrates (150). Animal experiments demonstrated type I IFN dependence on SS development in female NOD mice and elevated pDC (the main producer of type I IFN) TYPE I IFN in their submandibular gland (SMG). After injection of pDCs consuming anti-BST2/CD317 antibodies into female NOD mice aged 4 to 7 weeks, the lack of pDCs hindered the development of SMG inflammation and secretion dysfunction and significantly reduced the number of TYPE I IFN mRNA, white blood cell count, and T and B lymphocytes in the SMG. The expression of IL-7, BAFF, TNF-α, IFN-γ, CXCL9, CXCL11, CD40, CD40 L, Lt-α, Lt-β and NOS2 decreased (92). A study confirmed that overexpression of both type I and type II interferon-induced genes (IFIG) was simultaneously observed in peripheral blood and MSG tissues of patients with pSS (13). Recent studies have suggested that type III IFN (also known as IFN-λ) may be involved in the pathogenesis of pSS. Epithelial IFN-λ2/IL-28a expression was increased in the MSGs of pSS patients compared with non-PSS controls (151). These results suggest the role of the IFN pathway in the pathogenesis of pSS.

#### 5.2.7 LAMP3/HSP70/BMP6

BMP6 is a central cytokine that induces pSS-related secretion dysfunction. BMP6 can inhibit the water permeability of the salivary gland epithelial cell membrane by downregulating aquaporin 5 (AQP5), while local overexpression of BMP6 in the salivary gland or lacrimal gland can lead to loss of body fluid secretion in mice (152). HSP70 is an endogenous natural TLR4 ligand (TLR4 is an upstream regulator of BMP6) that stimulates BMP6 expression in pSS. The release of HSP70 from salivary epithelial cells may be triggered by overexpression of lysosome-associated membrane protein 3 (LAMP3). RT−PCR of small salivary gland RNA in pSS patients confirmed a positive correlation between BMP6 and LAMP3 expression. However, LAMP3 overexpression can induce BMP6 expression and a pSS phenotype in murine monocytes. The newly discovered LAMP3/HSP70/BMP6 axis provides an etiological model for SS gland dysfunction and autoimmunity (153).

#### 5.2.8 Pro-resolving mechanism

Salivary gland inflammation in pSS is generally triggered by viral and bacterial infections in susceptible individuals, leading to initial tissue loss. Neutrophils and M2 macrophages clear the site of injury or infection when the decomposition mechanism works appropriately (154). However, when this mechanism is abnormal, dead cells are not cleared in time, leading to the formation of their antigens, increased levels of cytokines and chemokines, and lymphocyte infiltration (155). Specific pro-resolving mediators (SPMs, including liposomes, resolvins, marisins, and protectin) and their aspirin-triggered (AT) forms act as inflammatory mediators, promoting tissue regeneration by limiting uncontrolled inflammation while promoting its termination (156). Odusanwo et al. found that the RvD1 receptor ALX/FPR2 was present in fresh, isolated salivary gland cells and salivary-derived cell lines of 16-week-old C57BL/6 mice in animal experiments. RvD1 receptor activation eliminates tight junctions and cytoskeletal disruption caused by TNF-α by modulating the phosphatidylinositol 3-kinase (PI3K)/AkT signaling pathway, enhances the migration and polarity of salivary epithelial cells, and promotes inflammation regression and tissue repair in salivary epithelial cells (157). Parashar et al. showed that the gene expression of enzymes involved in SPM biosynthesis was changed in the submandibular glands of NOD/ShiLtJ female mice, in which 5-LOX and 12/15-LOX were downregulated and upregulated, respectively. Specific predecomposition mediator (SPM) lysosomal D1 (RvD1) promotes the breakdown of salivary gland inflammation, and mice lacking the RvD1 receptor ALX/FPR2 exhibit congenital and adaptive immune deficiencies in salivary glands. Female ALX/FPR2 KO mice showed increased autoantibody production and loss of salivary gland function with age. This suggests that underlying SPM maladjustment may lead to SS progression (158).

## 6 The role of key inflammatory factors

### 6.1 TNF

In the salivary glands of patients with Sjogren’s syndrome, upregulated TNF-α induces apoptosis of epithelial cells and disrupts barrier function controlled by tight junction proteins such as the Claudin superfamily, resulting in reduced salivary secretion and gland atrophy (159). TNF-induced apoptosis occurs through the binding of TNF type I receptors (TNFR1), which contain death domains that transmit apoptotic signals through caspase activation (160). In addition, TNFα significantly increased the levels of caspase 3, 8, 9 and cytochrome C, leading to a decrease in the level of Bcl-2 and induced apoptosis of SMG-C6 cells and human SMG tissues (161). Caspase 3 is considered the most important executor of apoptosis, and caspase 8 initiates the death receptor pathway of apoptosis. Caspase 9 is a key player in the mitochondrial pathway and is involved in various stimuli. Cytochrome C is released from damaged mitochondria and plays a key role in inducing apoptosis. miRNAs regulate the expression of target genes at the posttranscriptional level, and many miRNAs are involved in the regulation of apoptosis. To determine the role of these miRNAs in TNFα -induced acinar cell apoptosis, real-time PCR was used to measure their expression levels after cells were incubated with TNFα for a specified period of time. The results showed that TNFα could induce significantly increased levels of Mir-34a-5p, Mir-34a-3p, Mir-200b-5p and Mir-200b-3p simultaneously, while leT-7a-5P expression remained unchanged (161).

### 6.2 Interleukin

Interleukin-2 (IL-2) and high-affinity IL-2 receptor (IL-2R) are essential for the survival of regulatory T cells (Tregs), which are major players in immune tolerance and prevention of autoimmune diseases. Elevated IL-2R levels were found to be positively correlated with SS severity, as reflected by pathologically low salivary flow. Due to the impaired IL-2/IL-2R signaling ability in pSS patients, the immunosuppressive function of Tregs in SS patients was weakened, which may induce salivary gland infiltration of lymphocytes and induce and aggravate pSS (162).

Recent studies have found that interleukin-6 is significantly higher in pSS patients than in HCs patients and is associated with mononuclear cell infiltration in salivary gland tissues in these patients (163). Salivary gland epithelial cells are the primary cellular source of increased IL-6 secretion in these patients. In addition, IL-6 can induce the transformation of SGECs from morphological and phenotypic to mesenchymal phenotypes in a dose-dependent manner. Recent studies have shown that IL-6-treated SGECs have decreased e-cadherin expression and increased vimentin and type I collagen expression compared to control cells. The results confirmed that IL-6 dysregulation may lead to EMT-dependent fibrosis (164).

IL-7 is a 25 kDa soluble globular protein produced and secreted by nonhematopoietic cells such as stromal cells, epithelial cells and endothelial cells. IL-7R is widely expressed in T and B cells, and IL-7/IL7R signaling is critical for the development and maintenance of the entire lymphoid compartment. *In vitro* experiments have shown that IL-7 induces the production of Th1- and Th2-related cytokines, including IFNγ, monocytes induced by IFNγ (MIG), IFNγ-inducible 10-KD protein (IP-10) and IL-4 (165). Another *in vitro* cell study showed that IL-7 stimulation induced higher IFN-γ, IL-4, IL-17 and IL-21 production in CCR9+ Th cells and CXCR5+ Th cells (166).

Katsifis et al. showed that the expression of IL-17 protein in salivary glands increased gradually with increasing biopsy lesion score. Transforming growth factor β, IL-6, and IL-23 are essential promoters of Th17 differentiation and are abundant compared to the amounts in control tissues (167). Animal experiments showed that IL-17 inhibited acetylcholine-induced calcium migration and downregulated transient receptor type 1 expression in SG epithelial cells by promoting Nfkbiz mRNA stabilization. In addition, local IL-17 neutralization in SGs significantly reduced salivation and improved tissue inflammation in mice (168). These results suggest that IL-17 may lead to salivary gland dysfunction in Sjogren’s syndrome by inhibiting TRPC1-mediated calcium movement.

IL-21, a member of the recently discovered type I cytokine family, is mainly secreted by Tfh and Tph cells. IL-21R is expressed in B cells and activated CD4+ T cells. IL-21 costimulates B cells with BCR to promote their differentiation into plasma cells, which is also necessary for the formation of normal germinal centers (GCs). The addition of IL-21 to the coculture system blocked 90% of B cells from differentiating into plasma cells. Animal experiments showed that IL-21R knockout mice completely eliminated spontaneous accumulation of GC B cells and plasma cells in blood (169). In addition, IL-21R is required for T_hef_ cell development. In general, IL-21R signaling is necessary for spontaneous accumulation of B and T-cell effector populations.

## 7 Treatment

As a target organ most frequently involved in pSS patients, the impairment of salivary gland function can lead to an imbalance in oral microecology and severe discomfort for patients. An increasing number of treatments are available with further research on the pathogenesis of pSS. In the following sections, we will review the treatment of pSS from the perspective of the immune microenvironment.

### 7.1 Resistance to hypoxia

As mentioned above, the salivary glands of pSS patients are chronically hypoxic, and blocking hypoxia development may be a potential treatment option. DMOG and FG-4497 hypoxic stabilizers have shown promising results in inflammatory bowel disease (IBD), reducing inflammation, reducing intestinal epithelial cell apoptosis, and enhancing intestinal barrier function (170–172). These may be potential drugs for improving salivary gland hypoxia. Moreover, drugs that inhibit PGE2, such as nonsteroidal drugs, may be equally effective in improving salivary gland function because of their role in hypoxia. In *in vitro* culture of human tubular HK-2 cells, cell death was mediated by COX-2-dependent PGE2 production, and the COX-2 inhibitor cefoxib prevented hypoxia-induced cell death (173). In another study of human retinal pigment epithelium, celecoxib also showed inhibition of HIF-1α under hypoxia (174)

### 7.2 Senolytics

Senescence makes salivary gland progenitor cells lose their ability to increase their value and differentiate, and the damaged salivary gland does not have enough regeneration potential to fully restore its function. Therefore, it is beneficial for pSS SGs to consume senescent cells and prevent the spread of senescence. Senolytics are a group of drugs that selectively eliminate senescent cells (175). In addition, pro-aging agents (such as navitoclax, dasatinib, and quercetin) work by inhibiting pro-survival pathways (such as Bcl-2 and Bcl-XL) to promote senescent cell death, thereby rejuvenating glandular cells and restoring glandular function (176). Selective removal of p16Ink4a-positive cells by ganciclovir or the antiaging drug ABT263 can eliminate senescent cells and improve the self-renewal ability of stem cells, thereby improving salivary gland function (177). Repressing cellular senescence contributes to the rescue of IR-induced hyposalivation by transient activation of Hh signaling, which is related to enhanced DNA repair and decreased oxidative stress in SMGs (178). Agonists of the Hh signaling pathway may be new targets for treating dryness. In addition, in an *in vitro* culture experiment of SGSCs after passage culture, the ROCK inhibitor Y-27632 inhibited the expression of senescence-related proteins and promoted cell proliferation (179). Another study showed that in C57BL/6 mice, loss of salivary function is closely related to cellular senescence, and radiation-induced loss of salivary gland function is dependent on IL-6, but IL-6 preconditioning can also prevent senescence and salivary gland hypofunction by enhancing DNA damage repair mechanisms (180). This suggests that IL-6 may play a dual role in Sjogren’s syndrome. A 6-month multicenter, double-blind, randomized placebo-controlled trial showed no improvement in systemic involvement and symptoms with toclizumab compared with placebo in patients with pSS (181). Currently, only toclizumab has been reported in the treatment of pSS with myelitis or refractory interstitial pneumonia (182, 183). However, the application of IL-6 in Sjogren’s syndrome remains to be explored.

### 7.3 Anti-inflammatory drugs

#### 7.3.1 Treatment of B cells and related factors

As a novel small molecule immunomodulator, iguratimod was confirmed to inhibit B cells by reducing immunoglobulin production and various inflammatory cytokines, including IL-1, IL-6, IL-8, and TNF (184). Clinical studies have validated that iguratimod improved some dryness symptoms and disease activity in pSS patients, reducing BAFF and the percentage of plasma cells over 24 weeks. It can also inhibit PGE2 production by selectively inhibiting COX-2 and the NFκB pathway (185). In animal studies, iguratimod improved inflammatory infiltration of the submandibular gland in mice (186).

Rituximab (RTX) is a monoclonal antibody that targets CD20 on B cells. CD20 is involved in the regulation of B lymphocyte growth after activation. In their open study using RTX, Carubbi et al. (179, 187) found that RTX treatment reversed specific focal lymphocytic sialoadenitis into a nonspecific chronic sialoadenitis mode by depleting B cells, resulting in complete recovery of small salivary gland structure in patients with residual SG function. However, other studies suggested that RTX anti-CD20 treatment might not deplete B-cell infiltration of pSS MALT sites (188). Gong et al. demonstrated in a mouse model that the local production of BAFF is a key local factor in MALt-mediated anti-RTX-depleting B cells (189). B-cell depletion can be achieved only when anti-BAFF is combined with anti-CD20.

Belimumab inhibits soluble BAFF. A one-year open-label trial on belimumab showed that the reduction in B-cell activation biomarkers observed at week 28 continued to week 52, but there was no change in salivary flow, Schirmer test, or salivary biopsy lesion scores (190). Immunobiological evidence supports a sequential regimen of RTX prebelimumab administration designed to target microenvironment BAFF first to improve the success rate of subsequent rituximab depletion therapy in MALT pathological tissues (189). Ianalumab is a monoclonal antibody that consumes B cells and blocks the B-cell activator receptor. In a double-blind, placebo-controlled phase II single-center study, ianalumab (VAY736) resulted in rapid and sustained B-cell depletion and improved ESSDAI and ESSPRI scores, but the variability in salivation flow rate was high enough to make any comparison difficult (191)

In pSS mouse models, labial gland mesenchymal stem cell-derived Exos (LGMSC-EXOS) reduced inflammatory infiltration and restored salivary secretion in salivary glands (192). LGMSC EXO-derived microRNA-125B affects the plasma cells of pSS by directly binding to its target gene, PRDM1 (PR domain zinc finger protein 1, also known as BLIMP1), which may be developed as a target gene for the treatment of pSS.

#### 7.3.2 Treatment of T cells and related factors

Cyclosporine A inhibits the IL-2 activity of T cells by interfering with calcineurin required for IL-2 gene transcription (193, 194). Hydroxychloroquine (HCQ) reduces the production of type I IFN and blocks the activation of TLR7 and TLR9 receptors (195), thereby interfering with antigen processing and blocking T-cell activation (196). However, in randomized, double-blind controlled trials in patients with pSS, HCQ did not improve disease symptoms despite inhibiting type I IFN-induced gene expression (188). The effect of HCQ alone on improving glandular function remains controversial.

Abatacept (CTLA4-Ig) binds to the costimulatory molecule CD80/CD86 and blocks the binding of these molecules to CD28 on T cells (197). A recent 48-week trial of abatacept in patients with pSS showed significant improvement in clinical and dry eye symptoms but not in stimulated whole salivary flow (198). Studies have shown a reduction in GCs in lymphocytic lesions and SG lip biopsies after abatacept treatment (199, 200), but salivary and lacrimal gland function remained stable (201).

In a recent clinical trial, prezalumab (a nondepleting monoclonal antibody against ICOSL) had a significant biological effect on SG inflammation, with a significant reduction in the number of CD4+ICOS+Tfh-like cells compared with placebo, despite the failure of the primary endpoint. demonstrated the biological efficacy of targeting the ICOS/ICOS-L pathway in pSS (202).

Other researchers have mitigated pSS by blocking MHC class II IAg7 antigen presentation in NOD mice to prevent pathogenic T cells from recognizing their antigens. The results showed that tetraazatricyclo-dodecane (TATD) and 8-azaguanine (8-AZA) alleviated symptoms by improving saliva and lacrimal gland secretion, reducing autoantibody levels, and reducing the severity of lymphocyte infiltration in saliva and lacrimal glands (203).

#### 7.3.3 Other anti-inflammatory drugs

Glucocorticoids are a widely used drug for chronic inflammatory autoimmune diseases. They bind to glucocorticoid receptors, resulting in increased transcription of anti-inflammatory genes, such as IL-10, and anti-inflammatory proteins that inhibit the expression of inflammatory genes. Studies have shown that glucocorticoid administration for 6 weeks improves saliva flow in patients but generally does not improve histological or functional parameters of SGs (204). However, a four-year long-term prospective study showed the opposite result: early pSS is characterized by a decline in salivary gland function, with or without steroid use, and a further decline in salivary gland function over time. Reduced salivary gland flow was not associated with corticosteroid use (205).

Leflunomide (LEF) inhibits pyrimidine biosynthesis and decreases naive and memory CD4+ T-cell and B-cell proliferation and NFκB activation (206, 207). In a phase II clinical trial involving 15 patients with early active PSS for 24 weeks, LEF treatment did not improve salivary flow (208). However, the combination of leflunomide and HCQ has been reported to increase salivary gland unstimulated significantly and stimulate total salivary production of pSS at certain time points (209, 210).

Drugs that treat pSS through the NF-κB signaling pathway, such as the novel synthetic DMARD drug iguratimod and the Syk signaling blocker GS-9876 (the Syk signaling pathway is upstream of IKK activation, and its blocking improves the release of NF-κB by its inhibitory complex), are currently in clinical trials. Their effect on the glands has yet to be tested.

In mouse models, Harim Tavares Dos Santos et al. found that hemolysin D1 (RvD1) and its aspirin-triggered AT-RVD1 effectively reduced inflammation and restored saliva flow before and after the onset of pSS. Resolvins are special proresolving mediators (SPMs) that can actively regulate inflammation. Furthermore, the expression of various SPM receptors (ALX/FPR2, BLT1, and CMKLR1) was found in human salivary glands, which may be a potential target for treating pSS patients (156).

CD40 is a transmembrane type I glycoprotein composed of 277 amino acids that belongs to the tumor necrosis factor (TNF) gene superfamily. The ligand CD40L/CD154 is a type II transmembrane protein and exists in a soluble (scd40L) or membrane-bound form. It is present on activated T cells, B cells, endothelial cells and epithelial cells (190). Compared with the control group, NOD mice treated with the CD40 DNA vaccine showed reduced lymphocyte infiltration and increased salivary secretion in salivary glands. At the same time, the expression levels of TNF-α and IL-6 in salivary glands decreased, the number of dendritic cells and plasma cells decreased, and the ANA level decreased (211). Iscalimab, an anti-CD40 antibody, has been shown to be safe and well tolerated at all doses in phase I clinical studies, with no clinically relevant changes in any of the safety parameters, including no evidence of thromboembolic events (212). However, its role in pSS patients remains to be further evaluated.

PSS patients have elevated levels of IL-7 and its receptor in salivary glands. Animal experiments showed that intraperitoneal injection of a blocking antibody against IL-7 receptor α chain (IL-7Rα) for 3 weeks in 10-week-old female NOD mice significantly improved characteristic SS pathology, including reduced salivary secretion and infiltration of leukocytes in the submandibular gland (SMG). Anti-IL-7r α treatment significantly reduced the amount of TNF-α in SMGs and increased the levels of Claudin-1 and aquaporin 5, two molecules essential for normal salivation (213). In phase I clinical trials of the monoclonal antibody GSK2618960 against interleukin-7 receptor α subunit (CD127), GSK2618960 was well tolerated and blocked IL-7 receptor signaling when fully targeted (214). This may be a new target for the future treatment of Sjogren’s syndrome.

## Conclusion

Oral salivary gland reduction is one of the most common clinical manifestations of pSS, a disease that directly affects exocrine function. The onset of the disease is genetically susceptible in cells, viruses, and other environmental factors under stimulation through chronic hypoxia, cell senescence, local inflammation, and the production of autoantibodies and other pathways in salivary gland cells, so that their function is impaired. This paper systematically reviews the characteristics and regulatory pathways of the salivary gland microenvironment, hoping that more targeted treatments can be developed to restore gland function and improve dry mouth symptoms through an in-depth understanding of the local immune microenvironment.

## Data availability statement

The original contributions presented in the study are included in the article/Supplementary Material. Further inquiries can be directed to the corresponding author.

## Author contributions

ZT wrote the manuscript. LW and XL participated in the modification. All authors contributed to the article and approved the submitted version.

## Funding

This article was supported by the National Natural Science Foundation of China (No. 81871271 and No. U21A20365), the Fundamental Research Funds for the Central Universities (grant number WK 9110000148).

## Conflict of interest

The authors declare that the research was conducted in the absence of any commercial or financial relationships that could be construed as a potential conflict of interest.

## Publisher’s note

All claims expressed in this article are solely those of the authors and do not necessarily represent those of their affiliated organizations, or those of the publisher, the editors and the reviewers. Any product that may be evaluated in this article, or claim that may be made by its manufacturer, is not guaranteed or endorsed by the publisher.
